# A nomogram to predict gestational diabetes mellitus: a multicenter retrospective study

**DOI:** 10.1093/jmcb/mjaf008

**Published:** 2025-03-10

**Authors:** Rui Zhang, Zhangyan Li, Nuerbiya Xilifu, Mengxue Yang, Yongling Dai, Shufei Zang, Jun Liu

**Affiliations:** Department of Endocrinology, Shanghai Fifth People's Hospital, Fudan University, Shanghai 200240, China; Department of Endocrinology, Shanghai Fifth People's Hospital, Fudan University, Shanghai 200240, China; Department of Endocrinology, Shanghai Fifth People's Hospital, Fudan University, Shanghai 200240, China; Endocrine Metabolism Department, The Second People's Hospital of Kashgar Prefecture, Kashi City 844000, China; Department of Endocrinology, Shanghai Fifth People's Hospital, Fudan University, Shanghai 200240, China; Endocrine Metabolism Department, The Second People's Hospital of Kashgar Prefecture, Kashi City 844000, China; Department of Endocrinology, Shanghai Fifth People's Hospital, Fudan University, Shanghai 200240, China; Department of Endocrinology, Shanghai Fifth People's Hospital, Fudan University, Shanghai 200240, China

**Keywords:** gestational diabetes mellitus, risk factors, nomogram

## Abstract

While gestational diabetes mellitus (GDM) poses great threat to the health of mothers and children, there is no standard early prediction model for this disease yet. This study developed and evaluated a nomogram for predicting GDM in early pregnancy. Overall, 1824 pregnant women were randomly divided into the training and internal validation sets in the ratio of 7:3, with additional 1604 pregnant women for external validation. Multivariate logistic regression analysis was used to develop a prediction model for GDM, and a nomogram was utilized for model visualization. Risk factors in the prediction model involved age, pre-pregnancy body mass index, reproductive history, family history of diabetes, creatinine level, triglyceride level, low-density lipoprotein level, neutrophil count, and monocyte count. Model performance was evaluated using receiver operating characteristic (ROC) curves, calibration curves, and decision clinical analysis (DCA). The area under ROC curve (AUC) value of the model was 0.804 for the training set, and similar AUC values were obtained for the internal (0.800) and external (0.829) validation sets, verifying the stability of the model. The calibration curves showed that the probabilities of GDM predicted by the nomogram highly correlated with the observed frequency values. The DCA curves indicated that the prediction model is clinically useful, thus potentially aiding early pregnancy management in women.

## Introduction

Gestational diabetes mellitus (GDM) is a common metabolic syndrome characterized by elevated blood glucose during pregnancy, which is now a clinical challenge because it highly increases adverse pregnancy outcomes (American Diabetes Association, 2020a). Moreover, GDM increases the risk of cesarean section, preeclampsia, and postpartum type 2 diabetes for pregnant women. It also heightens the risk of adverse pregnancy outcomes for offspring and contributes to increased rates of obesity and metabolic syndrome during adolescence (Sweeting et al., 2022). Among various diagnostic criteria for GDM, the most commonly used diagnosis is a single oral glucose tolerance test (OGTT) during 24–28 weeks of gestation. In the absence of a methodology for accurate risk assessment during early pregnancy, early screening and intervention for those at risk for GDM becomes difficult. Several studies have evaluated models for predicting GDM during early pregnancy, but predictive results vary across studies and ethnicities and remain generally unsatisfactory (Jokelainen et al., 2020; Liu et al., 2020; Nakanishi et al., 2020; Kang et al., 2021; Wang et al., 2021; Wu et al., 2021, 2023). Therefore, it is crucial to explore more accurate and practical prediction models.

The etiology of GDM is notably complex, primarily characterized by insulin resistance and β-cell dysfunction in its pathogenesis. In recent years, the idea that GDM is a chronic low-grade inflammation with a chronic metabolic response, diminishing insulin sensitivity and β-cell integrity, has gained wide acknowledegment. Furthermore, it has been shown that inflammatory blood cell parameters such as elevated white blood cell (WBC) and neutrophil counts, as well as neutrophil/lymphocyte ratio, can often be used as simple markers of inflammation (Wolf et al., 2004; Pantham et al., 2015; Yang et al., 2015). Previous studies in our group have demonstrated that elevated neutrophils and decreased monocytes in early pregnancy are strongly associated with gestational diabetes (Sun et al., 2020; Huang et al., 2022). Therefore, we suspected that neutrophil and monocyte counts may be potential predictors for GDM.

**Figure 1:**
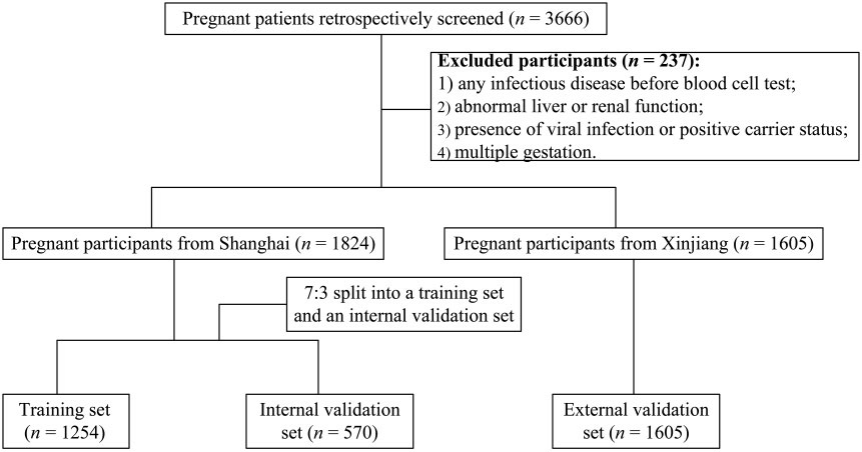
Flowchart of this study.

Additionally, multiple risk factors for GDM have been reported, including age, pre-pregnancy body mass index (BMI), lipid metabolism disorders, and biochemical markers. Unfortunately, to date, clinicians still lack a standardized model for early pregnancy screening to identify individuals at high risk for GDM. Therefore, we designed a multicenter retrospective cohort study (Figure 1) to construct a model for the prediction of GDM in early pregnancy and made a clinical practice nomogram.

## Results

### Patient characteristics

Table 1 shows the demographic and metabolic characteristics of the 3429 participants without a history of GDM included in this study (Figure 1). A total of 1824 cases from Shanghai were screened retrospectively, which were divided into a training set (*n* = 1254) and an internal validation set (*n* = 570) at an approximate ratio of 7:3. The baseline characteristics and prevalence of GDM were well-balanced, without significant differences between the training and internal validation sets (Table 1). A total of 1605 pregnant women recruited from another center (The Second People's Hospital of Kashgar Prefecture, Xinjiang) were included as the external validation set, in which the proportion of GDM was 25.5% (Table 1). The characteristics of the patients in the training, internal validation, and external validation sets are shown in Supplementary Tables S1–S3.

**Table 1:** Characteristics of the training and validation sets.

Variable	Training set	Internal validation set	External validation set
*n*	1254	570	1605
Age (years)	29 ± 5	29 ± 5	29 ± 4
GDM (*n*, %)	308 (24.6%)	134 (23.5%)	409 (25.5%)
Parity (*n*, %)			
Nulliparous	323 (25.8%)	148 (26.0%)	947 (59.0%)
Parous	931 (74.2%)	422 (74%)	658 (41.0%)
Family history of DM (*n*, %)			
No	1224 (97.6%)	561 (98.4%)	1509 (94.0%)
Yes	30 (2.4%)	9 (1.6%)	96 (6.0%)
Pre-pregnancy BMI (kg/m^2^)	22.3 ± 3.4	22.5 ± 3.5	22.3 ± 4.3
SBP (mmHg)	116 ± 11	117 ± 12	112 ± 10
DBP (mmHg)	70 ± 8	70 ± 9	71 ± 8
FBG (mmol/L)	4.58 ± 0.35	4.60 ± 0.34	4.40 ± 0.34
ALT (U/L)	13.0 (9.0–18.3)	13.0 (10.0–19.0)	13.0 (9.8–19.9)
Cr (mmol/L)	45 ± 9	44 ± 8	45 ± 8
UA (µmol/L)	210 (189–229)	212 (189–229)	223 (196–266)
TG (mmol/L)	1.80 (1.32–2.49)	1.82 (1.36–2.38)	2.07 (1.57–2.80)
CH (mmol/L)	4.94 ± 1.10	4.90 ± 0.98	5.31 ± 1.22
HDL (mmol/L)	1.81 ± 0.38	1.81 ± 0.38	1.73 ± 0.42
LDL (mmol/L)	2.53 ± 0.80	2.51 ± 0.78	2.76 ± 0.80
WBC (×10^9^/L)	8.71 ± 1.97	8.77 ± 2.10	8.59 ± 2.32
Neutrophil (×10^9^/L)	6.26 ± 1.76	6.25 ± 1.76	6.08 ± 1.92
Lymphocyte (×10^9^/L)	1.75 ± 0.47	1.72 ± 0.47	1.84 ± 0.75
Monocyte (×10^9^/L)	0.50 ± 0.24	0.50 ± 0.21	0.55 ± 0.21
Platelet (×10^9^/L)	222 ± 52	222 ± 51	248 ± 64
Hemoglobin (g/L)	122 ± 10	122 ± 11	120 ± 15

Data are mean ± SD, *n* (%), or median (IQR).

ALT, alanine aminotransferase; CH, cholesterol; Cr, creatinine; DM, diabetes mellitus; FBG, fasting blood glucose; HDL, high-density lipoprotein; SBP, systolic blood pressure; UA, uric acid.

### Univariable analysis and multivariable analysis

In the training set, we first screened variables with *P*-values ˂0.1 by performing univariable logistic regression analysis for inclusion in multivariable analysis. These variables included age, parity, family history of diabetes, pre-pregnancy BMI, diastolic blood pressure (DBP), creatinine level, uric acid, triglyceride (TG) level, low-density lipoprotein (LDL) level, and WBC, neutrophil, monocyte, hemoglobin, and platelet counts (Table 2). However, WBC was excluded from subsequent analysis because of its close correlation with neutrophils and monocytes. Finally, factors that maintained notable associations with GDM in multivariable logistic regression and were clinically relevant to GDM, including age, parity, family history of diabetes, pre-pregnancy BMI, creatinine level, TG level, LDL level, and neutrophil and monocyte counts, were selected to construct a prediction model (Table 2). The variation inflation factors (VIFs) of these variables in the training set were ˂2, indicating no significant collinearity.

**Table 2:** Univariable and stepwise multivariable logistic analysis of risk factors for GDM.

	Univariable analysis			Multivariable analysis		
	β	OR (95% CI)	*P*	β	OR (95% CI)	*P*
Age (years)	0.101	1.106 (1.077–1.137)	<0.001	0.091	1.095 (1.062–1.130)	<0.001
Parity (*n*, %)						
Nulliparous		Reference			Reference	
Parous	−0.809	0.445 (0.338–0.588)	<0.001	−1.052	0.349 (0.245–0.498)	<0.001
Family history of DM						
No		Reference			Reference	
Yes	2.199	9.019 (3.973–20.47)	<0.001	1.785	5.957 (2.322–15.286)	<0.001
Pregnancy BMI (kg/m^2^)	0.138	1.148 (1.106–1.192)	<0.001	0.111	1.118 (1.071–1.166)	<0.001
SBP (mmHg)	0.006	1.006 (0.994–1.018)	0.331		Not selected	
DBP (mmHg)	0.016	1.016 (0.998–1.031)	0.044		−	
FBG (mmol/L)	−0.072	0.931 (0.646–1.341)	0.700		Not selected	
ALT (U/L)	0.018	1.018 (1.006–1.030)	0.004		−	
Cr (mmol/L)	0.035	1.036 (1.021–1.051)	<0.001	0.024	1.025 (1.006–1.044)	0.010
UA (µmol/L)	0.007	1.007 (1.004–1.011)	<0.001		−	
TG (mmol/L)	0.337	1.401 (1.247–1.575)	<0.001	0.391	1.479 (1.282–1.705)	<0.001
CH (mmol/L)	−0.053	0.949 (0.833–1.080)	0.425		Not selected	
HDL (mmol/L)	−0.257	0.774 (0.552–1.085)	0.137		Not selected	
LDL (mmol/L)	0.505	1.658 (1.409–1.950)	<0.001	0.555	1.742 (1.440–2.107)	<0.001
WBC (×10^9^/L)	0.135	1.145 (1.073–1.221)	<0.001		Not selected	
Neutrophil (×10^9^/L)	0.256	1.292 (1.200–1.391)	<0.001	0.306	1.358 (1.229–1.499)	<0.001
Lymphocyte (×10^9^/L)	0.007	1.007 (0.765–1.325)	0.961		Not selected	
Monocyte (×10^9^/L)	−0.717	0.488 (0.250–0.952)	0.035	−2.846	0.058 (0.017–0.195)	<0.001
Platelet (×10^9^/L)	0.002	1.002 (1.000–1.005)	0.049		−	
Hemoglobin (g/L)	0.016	1.016 (1.003–1.029)	0.016		−	

### Development and validation of the nomogram to predict GDM

**Figure 2:**
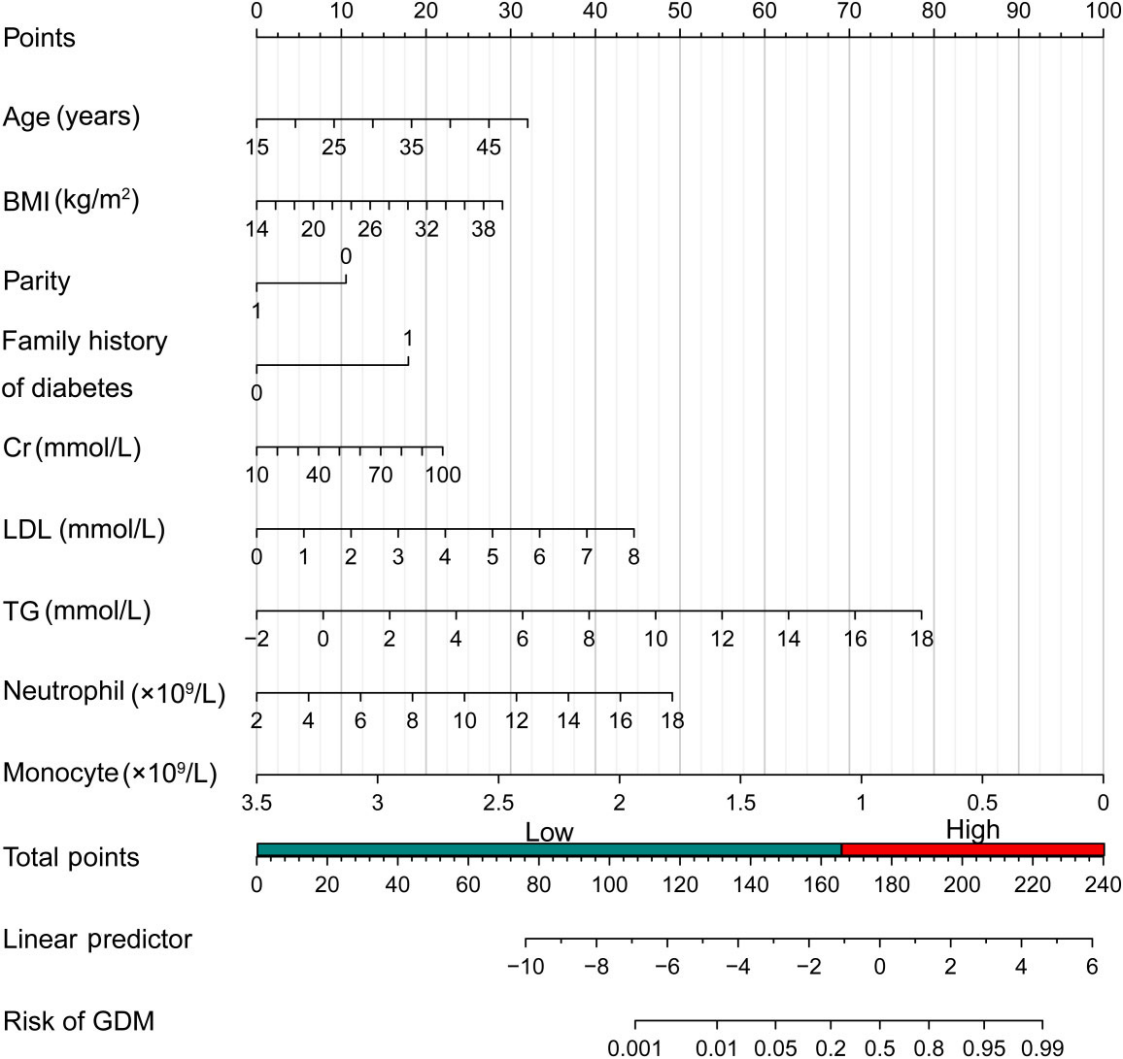
Nomogram of the prediction model to estimate the risk of GDM. Once a patient's variables are available, the position of each variable is determined on its axis, and a vertical line is drawn from each position to the ‘Points’ axis. The total points can be calculated by adding the points of all variables. Then, a vertical line is drawn from the ‘Total points’ axis to the ‘Risk of GDM’ axis to obtain the predicted risk value. For example, a 33-year-old pregnant woman with a BMI of 24.2 kg/m^2^, nulliparous, no family history of diabetes, a creatinine (Cr) level of 51 mmol/L, an LDL level of 3.09 mmol/L, a TG level of 1.27 mmol/L, a neutrophil count of 4.25 × 10^9^/L, and a monocyte count of 0.40 × 10^9^/L would have 174 total points and a risk of developing GDM of 43% based on this nomogram.

**Figure 3:**
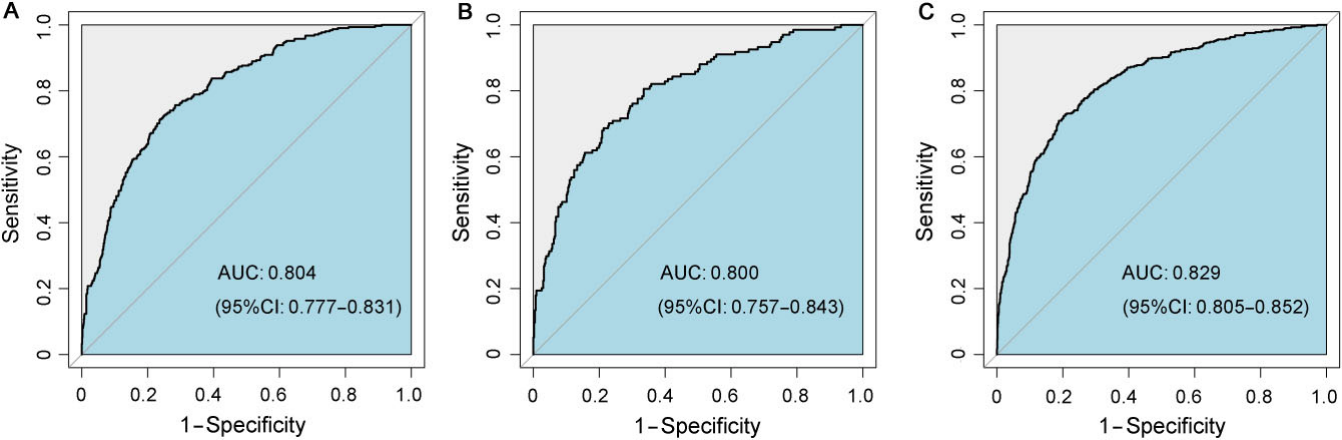
ROC curves of the prediction model for the training (**A**), internal validation (**B**), and external validation (**C**) sets.

**Figure 4:**
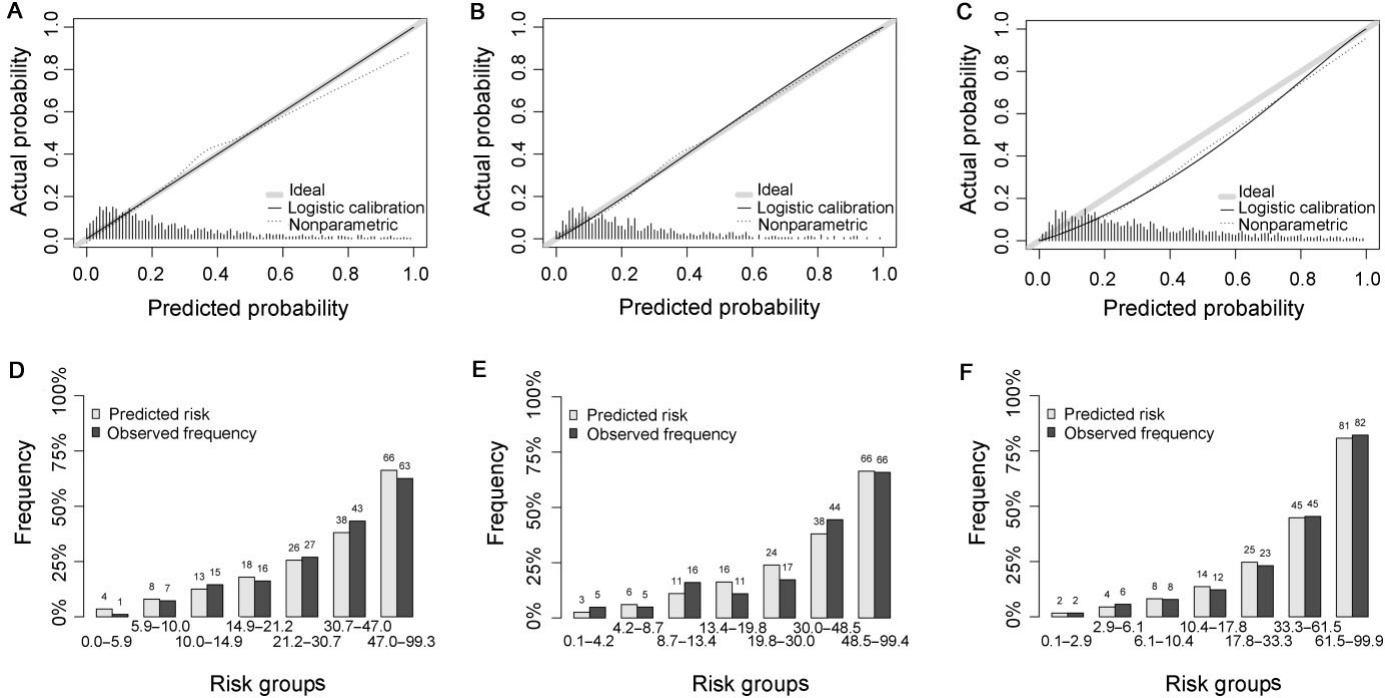
Calibration curves of the GDM nomogram in the training (**A**), internal validation (**B**), and external validation (**C**) sets. The predicted and actual probabilities of GDM were divided into seven groups for the training (**D**), internal validation (**E**), and external validation (**F**) sets.

Based on the results from the logistic regression analysis and risk factor calculation in the training set, a nomogram was constructed using the *P*-values and effect values of the nine variables (Figure 2). The area under receiver operating characteristic (ROC) curve (AUC) values of the prediction model were 0.804 (95% CI: 0.777–0.831) in the training set, 0.800 (95% CI: 0.757–0.843) in the internal validation set, and 0.829 (95% CI: 0.805–0.852) in the external validation set, indicating a good discrimination of the model (Figure 3). The calibration curves showed high correlation between the predicted and observed probabilities of GDM in the training, internal, and external validation sets (Figure 4A–C). Bar charts were created by dividing the predicted and actually observed probabilities of GDM into seven groups, further indicating a good match between the predicted risk and observed frequency of GDM in the training, internal validation, and external validation sets (Figure 4D–F). Both the Brier score of 0.143 and the F1 score of 0.822 indicated the good overall performance of the model (Model 3) in the training set, and favorable Brier and F1 scores were also obtained in the validation sets (Table 3).

**Table 3:** Evaluation of discrimination and calibration abilities of the models.

Model	Brier score	AUC (95%)	Discrimination index	Quality index	F1 score
**Model 1: Age + BMI + DM + parity**					
Training set	0.161	0.729	0.125	0.127	0.774
Internal validation set	0.149	0.760	0.160	0.155	0.767
External validation set	0.151	0.788	0.188	0.154	0.661
**Model 2: Age + BMI + Cr + LDL + TG + DM + parity**					
Training set	0.149	0.781	0.194	0.196	0.740
Internal validation set	0.145	0.770	0.185	0.188	0.733
External validation set	0.157	0.803	0.165	0.087	0.490
**Model 3: Age + neutrophils + BMI + monocyte + Cr + LDL + TG + DM + parity**					
Training set	0.143	0.804	0.233	0.235	0.822
Internal validation set	0.137	0.800	0.229	0.232	0.826
External validation set	0.141	0.829	0.253	0.218	0.737
External validation subset (parity)					
Nulliparous	0.133	0.852	0.181	0.068	0.703
Parous	0.151	0.830	0.321	0.322	0.788

### Comparison of the performance of the GDM model represented by the nomogram with those constructed from different combinations of variables

To identify the optimal model, three models were constructed from different combinations of variables (Model 1 included age, parity, family history of diabetes, and pre-pregnancy BMI; Model 2 included age, parity, family history of diabetes, pre-pregnancy BMI, and creatinine, TG, and LDL levels; Model 3 included all nine variables and was represented by a nomogram). As shown in Table 3, Model 3 with the Brier score of 0.143, AUC of 0.804, and F1 score of 0.822 showed the best overall performance. The discrimination and quality indices of Model 3 were better than those of Model 1 and Model 2. Considering that more sophisticated algorithms may offer additional insights and potentially improve predictive accuracy, we also constructed a model using the Random Forest algorithm in machine learning and evaluated its performance (Supplemental Tables S6 and S7).

### Comparison of patient characteristics and incidence of GDM between low-risk and high-risk groups

Based on the nomogram, each patient's total points were calculated, and Youden's index of the ROC curve was used to determine the optimal cut-off value, e.g. 166 points in the training set (Supplementary Figure S1). Accordingly, patients were stratified into two risk groups (high-risk vs. low-risk) in the training, internal validation, and external validation sets, respectively (Supplementary Tables S1–S3). The participants in the high-risk group showed a higher incidence of GDM than those in the low-risk group (Supplementary Table S4).

### Clinical usefulness of the nomogram

**Figure 5:**
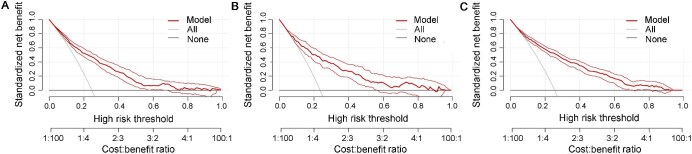
DCA curves of GDM nomogram for the training (**A**), internal validation (**B**), and external validation (**C**) sets. Solid line: assuming that no patients are treated, and the net benefit is zero; gray line: assuming that all patients are treated; red line: patients are treated if the proposed model exceeds a threshold. For example, in the internal and external validation sets, if the threshold (predicted probability of GDM) is between 10% and 70%, it can be beneficial (positive net benefit) for making GDM diagnostic decisions. However, if the probability of GDM is <10% or >70%, there is no additional benefit for diagnosing GDM.

To assess the clinical utility of the nomogram, decision clinical analysis (DCA) curves were constructed for the training, internal validation, and external validation sets. Positive net benefits were achieved at predicted probability thresholds 0.1–0.7, indicating that further diagnosis is beneficial when the predicted risk value is between 10% and 70% (Figure 5).

### Subgroup analysis

Although the model had a favorable AUC value for external validation, the predicted probabilities were higher than the actually observed probabilities (Figure 4C). Since the percentage of nulliparous individuals in the external validation set was found to be higher than that in the training set (Supplementary Table S3), participants in the external validation set were further divided into nulliparous and parous subgroups. Notably, the nomogram showed better performance in the parous subgroup than in the nulliparous group (Table 3, F1 score 0.788 vs. 0.703; Supplementary Figure S2).

## Discussion

In this study, a prediction model was developed to estimate the probability of pregnant women developing GDM by incorporating demographic characteristics, serum biochemical markers, and inflammatory cell counts during early pregnancy. This model was internally and externally validated, and a predictive nomogram for GDM was subsequently established. Furthermore, the prediction model was demonstrated to exhibit a good predictive performance.

Medical nutrition therapy and weight management are crucial for the early treatment of gestational diabetes. Studies have shown that 70%–85% of women diagnosed with GDM can improve their blood glucose status by changing their lifestyle (American Diabetes Association, 2017, 2020b; Yamamoto et al., 2018; Rasmussen et al., 2020). Timely warning and intervention during early pregnancy play key roles in the prevention and treatment of GDM. Therefore, early prediction tools are crucial in high-risk women. To date, clinicians rely on a subset of prediction models based on demographic characteristics and serum biochemical markers to aid in decision-making (Perry et al., 2014; Schoenaker et al, 2018; Li et al., 2020, 2023; Wu et al., 2021). However, these models have several limitations in clinical applications. For example, a lower AUC value affects the predictive performance of the model. Models that include too many variables may result in overfitting and are unsuitable for widespread use. Additionally, variables that are not readily available can make the model inconvenient for clinical applications. Our model exhibited a good predictive performance, with an AUC value of 0.804. This was achieved by using nine common and easily accessible predictors. We also constructed and compared different models to determine that the model represented by the nomogram was optimal.

Although the use of logistic regression in our study is indeed a common approach, we also compared the performance of logistic regression with that of machine learning algorithms (Random Forest) and found that the machine learning method is not particularly superior to the model established by logistic regression. There are several reasons why logistic regression might be considered more suitable or superior to machine learning methods: (i) logistic regression provides coefficients that are directly interpretable in terms of the log-odds of the outcome, which is crucial for clinical decision-making and generating new hypotheses; (ii) the assumptions and the mathematical formulation are well-understood, making it easier for other researchers and clinicians to replicate and validate the findings; and (iii) machine learning models can be prone to overfitting when the sample size is not large enough relative to the number of model parameters.

The clinical parameters included in our algorithm were chosen due to their established relevance in the clinical literature and their potential to influence GDM. Our team consists of experienced clinicians who have extensive knowledge in the field. Thus, the chosen parameters reflect their insights into which factors are most likely to be predictive of the clinical outcome. Age, BMI, and family history of diabetes are well-known risk factors for GDM, and these indicators are readily available to be included. Since pregnant women undergo routine obstetric examinations in early pregnancy, routine biochemical indicators such as creatinine, TG, and LDL are also readily available to be included in the model. Moreover, studies have shown that neutrophils, monocytes, and inflammatory cells in the body are implicated in low-grade chronic inflammation, which plays a crucial role in the pathophysiology of GDM and type 2 diabetes mellitus (Kuzmicki et al., 2008; Dalfrà et al., 2009; Syngelaki et al., 2016). Our prediction model incorporates easily available parameters such as neutrophils and monocytes, and thus the prediction model is cost-effective. We are aware that the addition of some potential influencing factors might enhance the model's performance. Therefore, we will explore the possibility of incorporating other relevant clinical parameters that were not initially included in our model.

Among the risk factors for the recurrence of gestational diabetes, a history of GDM has been reported as a risk factor (Kim et al., 2007; Zhang et al., 2013, 2023; Sweeting et al., 2022). Pregnant women with a history of GDM should closely monitor their glucose levels during pregnancy and take early interventional measures to prevent the recurrence of GDM. This model excluded participants with a history of GDM and was particularly valuable in predicting the risk of pregnant women developing GDM for the first time.

After participants were divided into nulliparous and parous subgroups in the external validation set, the calibration curves validated the consistency between the predicted and observed values in the parous subgroup, suggesting that the model could make more accurate predictions in parous pregnant women for widespread use.

This study has several advantages. First, a large-sample multicenter study was conducted at a separate center for external validation, and the performance of the model was robust during external validation, indicating a high extrapolation. Second, our model is relatively simple, with predictive parameters that are easy and inexpensive to obtain.

However, this study also has limitations. First, the prediction model is a short-term preventive predictor of gestational diabetes, while long-term cohort studies are needed to explore prediction models for the development of diabetes after pregnancy. Second, although we considered the potential GDM risk factors, the possibility of residual confounders could not be excluded.

In conclusion, the present study offers a simple and reliable prediction model for identifying pregnant women at high risk of GDM, demonstrating good discrimination. Furthermore, our prediction model shows the importance of conducting randomized controlled trials to assess the impact of early intervention on high-risk women.

## Materials and methods

### Study design and participants

Our study included 3429 singleton pregnant women without a history of GDM. Among them, 1824 pregnant women were recruited from the Obstetrics Department of Shanghai Fifth People's Hospital, Fudan University, between February 2018 and June 2022. The Shanghai study dataset was divided into the training and internal validation sets (∼7:3) to ensure comparability. As an external validation set, 1605 pregnant women were recruited from the Department of Obstetrics at The Second People's Hospital of Kashgar Prefecture, Xinjiang, between March 2019 and May 2023. The procedure used in this retrospective study is shown in Figure 1.

GDM was diagnosed if at least one of the following thresholds is reached in the 2-h 75-g OGTT: fasting plasma glucose ≥5.1 mmol/L, 1-h plasma glucose ≥10.0 mmol/L, or 2-h plasma glucose ≥8.5 mmol/L (International Association of Diabetes and Pregnancy Study Groups Consensus Panel, 2010).

### Data collection

The clinical and laboratory data of pregnant women were retrospectively collected from 8–12 weeks of gestation. Pregnancy data collection and laboratory evaluation methods have been previously reported (Sun et al., 2020).

### Statistical analyses

Descriptive statistics of the variables are presented as mean ± standard deviation (SD) for normally distributed variables, median, and interquartile range (IQR) for non-normally distributed variables, and frequencies and proportions for categorical variables. The participants from Shanghai were randomly divided into the training and internal validation sets at a 7:3 ratio. Univariate logistic regression was used to screen for relevant risk factors. Odds ratios (ORs) were calculated to estimate the strength of the association between risk factors and GDM. In the training set, variables with *P*-values ˂0.1 and clinically recognized risk factors for GDM were selected for inclusion in the multivariate logistic regression model, using the stepwise regression method with the backward approach. VIF was used to assess the collinearity of various combinations of variables in the training set. The Brier score was used to determine overall performance, ranging from 0 (excellent prediction) to 1 (worst prediction), which is a reconciled average of precision and recall. The AUC value was calculated to assess the discriminatory power of the model. Calibration curves were used to study the agreement between predicted and actual observations. The clinical benefits of the model were analyzed using DCA. Finally, visual nomograms were obtained. External validation was performed using data from Xinjiang.

All statistical analyses were performed using SPSS software (version 25.0; IBM SPSS) and R software version 4.0.2. The major R software packages used in this study are shown in Supplementary Table S5. All *P*-values were two-tailed, and statistical significance was set at *P* < 0.05.

### Ethics declarations

This study was approved by the Ethics Committee of Shanghai Fifth People's Hospital, Fudan University (2020154). Informed consents were obtained from patients or their representatives, and the protocol conformed with the Declaration of Helsinki.

## Supplementary Material

mjaf008_Supplemental_File

## Notes

**Author contributions:** R.Z.: clinical data collection, formal analysis, and writing the original draft. Z.L.: clinical data collection, data analysis, and writing—revised manuscript. N.X.: data analysis, supervision, and writing—review and editing. M.Y.: statistical analysis. Y.D.: clinical data collection. S.Z.: subject design and supervision. J.L.: conceptualization, supervision, and writing—review and editing. All authors read and approved the final manuscript.
