# Supplementary material for: A nomogram to predict gestational diabetes mellitus: a multicenter retrospective study
Source: J Mol Cell Biol. 2025 Mar 10;17(3):mjaf008. doi: 10.1093/jmcb/mjaf008 (PMC12448233; doi:10.1093/jmcb/mjaf008)
Supplement: mjaf008_Supplemental_File [file mjaf008_supplemental_file.pdf]

Supplementary material

A nomogram to predict gestational diabetes mellitus: a multi-center retrospective study

Rui Zhang[1,†], Zhangyan Li[1,†], Nuerbiya Xilifu[1,2], Mengxue Yang[1], Yongling Dai[2], Shufei Zang[1,*], and Jun Liu[1,*]

[1] Department of Endocrinology, Shanghai Fifth People's Hospital, Fudan University, 801 Heqin Road, Shanghai 200240, China

[2] Endocrine Metabolism Department, The Second People's Hospital of Kashgar Prefecture, No. 1, Health Road, Kashi City, Kashi Prefecture, Xinjiang Uygur Autonomous Region, 844000, China

[†] These authors contributed equally to this work.

* Correspondence to: Jun Liu, E-mail: liu__jun@fudan.edu.cn; Shufei Zang, E-mail: sophiazsf@fudan.edu.cn, Tel: +86-02164308151, Fax: +86-02164308151

Running title: Nomogram for GDM Prediction

Supplementary Figures

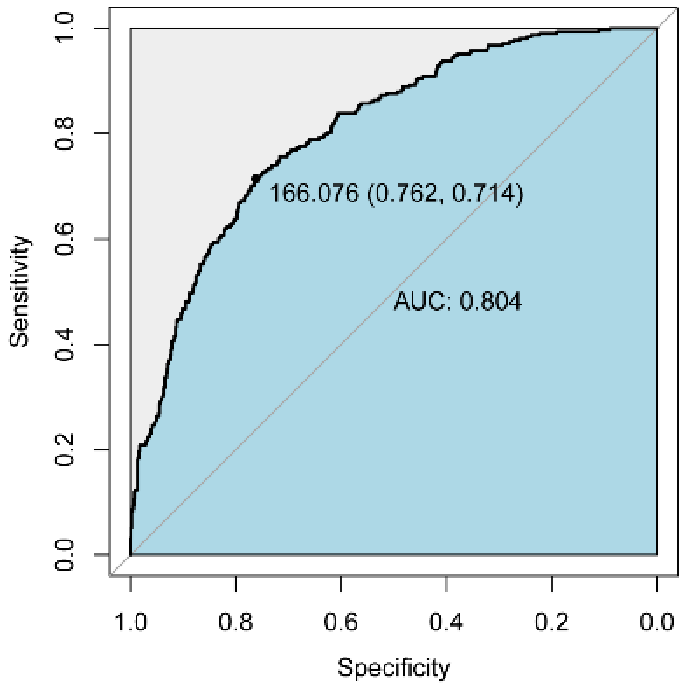

Supplementary Figure 1 ROC curve and optimal cutoff point for risk score in the training set

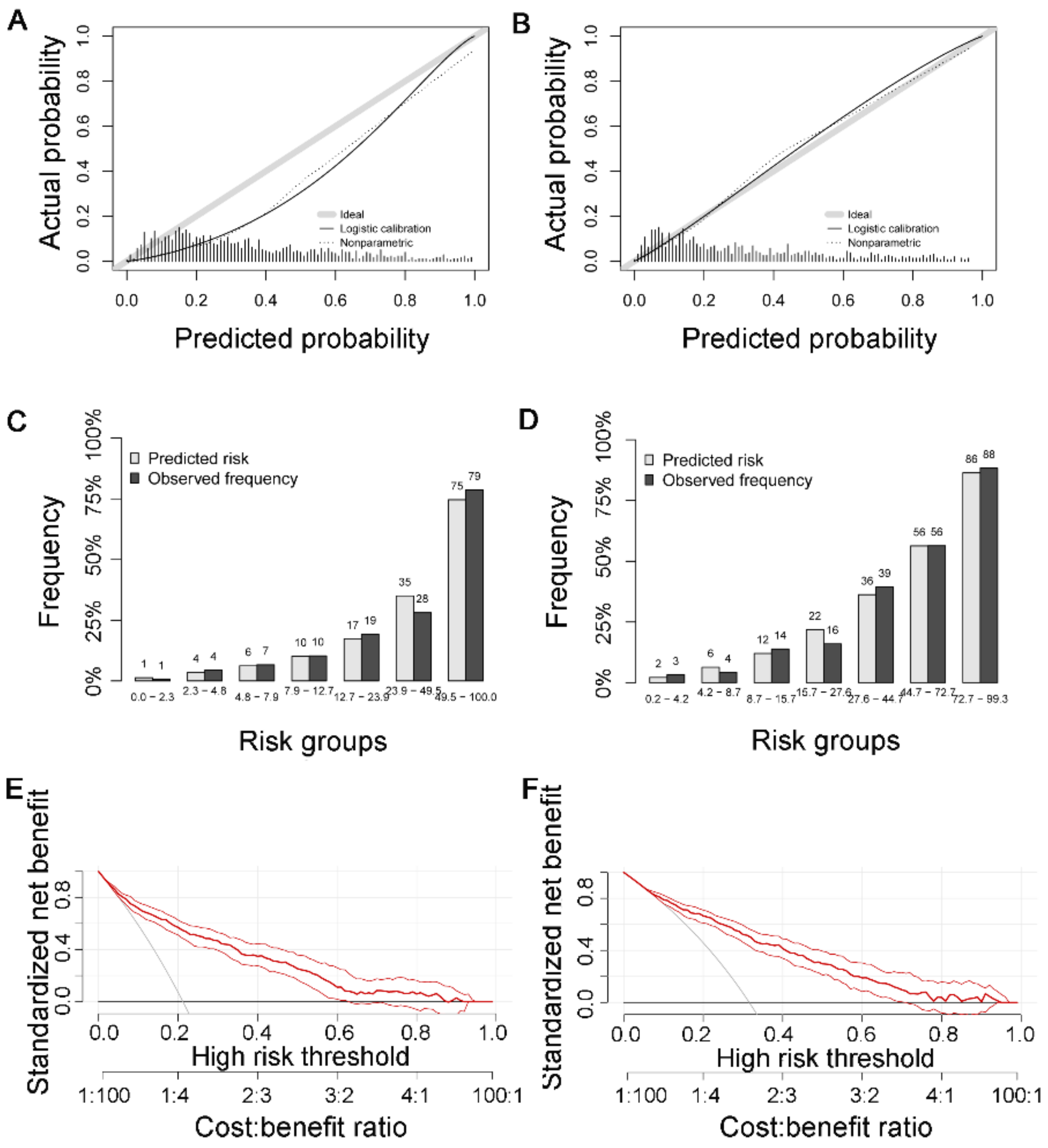

Supplementary Figure 2 Calibration curves and decision curves analysis of GDM nomogram of nulliparous (ACE) and parous (BDF) subgroups in the external validation set.

Supplementary Tables

Supplementary Table S1 Characteristics of the Training Set.

Variable	Women without GDM	Women with GDM	*P*	Low-risk participants	High-risk participants	*P*
n	946	308		809	445	
Age (years)	28 ± 5	30 ± 5	<0.001	27 ± 4	31 ± 5	<0.001
Parity (n, %)						
Nulliparous	205 (21.7%)	118 (38.3%)	<0.001	134 (16.6%)	189 (42.5%)	<0.001
Parous	741 (78.3%)	190 (61.7%）		675 (83.4%)	256 (57.5%)	
Family history of DM (n, %)						
No	938 (99.2%)	286 (92.9%)	<0.001	808 (99.9%)	416 (93.5%)	<0.001
Yes	8 (0.8%)	22 (7.1%)		1 (0.1%)	29 (6.5%)	
Pre-pregnancy BMI (kg/m^2)	21.9 ± 3.2	23.6 ± 3.7	<0.001	21.4 ± 2.7	24.1 ± 3.8	<0.001
SBP (mmHg)	116 ± 11	117 ± 10	0.327	116 ± 10	119 ± 11	<0.001
DBP (mmHg)	70 ± 8	71 ± 8	0.043	69 ± 8	71 ± 9	<0.001
FBG (mmol/L)	4.58 ± 0.35	4.57 ± 0.37	0.700	4.59 ± 0.35	4.56 ± 0.36	0.128
ALT (U/L)	13.0 (9.0–18.0)	13.0 (9.5–22.0)	0.068	12.0 (9.0–17.0)	14.0 (10.0–22.0)	<0.001
Cr (mmol/L)	44 ± 9	47 ± 8	<0.001	43 ± 8	48 ± 9	<0.001
UA (μmol/L)	207 (189–229)	216 (193–238)	<0.001	206 (189–229)	216 (190–235)	<0.001
TG (mmol/L)	1.66 (1.24–2.36)	1.96 (1.65–2.88)	<0.001	1.61 (1.22–2.18)	2.08 (1.59–3.02)	<0.001
CH (mmol/L)	4.96 ± 1.08	4.89 ± 1.16	0.441	4.75 ± 0.98	5.27 ± 1.20	<0.001
HDL (mmol/L)	1.82 ± 0.39	1.78 ± 0.36	0.137	1.81 ± 0.39	1.81 ± 0.38	0.943
LDL (mmol/L)	2.45 ± 0.77	2.78 ± 0.84	<0.001	2.34 ± 0.70	2.88 ± 0.85	<0.001
WBC (×10^9/L)	8.59 ± 1.94	9.12 ± 2.00	<0.001	8.41 ± 1.89	9.28 ± 1.99	<0.001
Neutrophils (×10^9/L)	6.06 ± 1.65	6.88 ± 1.92	<0.001	5.85 ± 1.53	7.01 ± 1.89	<0.001
Lymphocyte (×10^9/L)	1.75 ± 0.47	1.75 ± 0.47	0.961	1.75 ± 0.48	1.76 ± 0.43	0.781
Monocyte (×10^9/L)	0.51 ± 0.27	0.48 ± 0.14	0.004	0.52 ± 0.28	0.47 ± 0.14	<0.001
Platelet (×10^9/L)	221 ± 52	227 ± 53	0.051	218 ± 52	230 ± 52	<0.001
Hemoglobin (g/L)	121 ± 10	123 ± 11	0.015	121 ± 10	124 ± 10	<0.001

Data are mean ± SD or n (%).

ALT, alanine aminotransferase; BMI, body mass index; CH, cholesterol; Cr, creatinine; SBP, systolic blood pressure; DBP, diastolic blood pressure; FBG, fasting blood glucose; GDM, gestational diabetes mellitus; HDL-C, high-density li*p*oprotein cholesterol; LDL-C, low-density lipoprotein cholesterol; TG, triglycerides; UA, uric acid; DM: diabetes mellitus.

Supplementary Table S2 Characteristics of the Internal Validation Set.

Variable	Women without GDM	Women with GDM	*P*	Low-risk participants	High-risk participants	*P*
n	436	134		375	195	
Age (years)	28 ± 4	31 ± 5	<0.001	28 ± 4	31 ± 5	<0.001
Parity (n, %)						
Nulliparous	93 (21.3%)	55 (41.0%)	<0.001	64 (17.1%)	84 (43.1%)	<0.001
Parous	343 (78.7%)	79 (59.0%)		311 (82.9%)	111 (56.9%)	
Family history of DM (n, %)						
No	433 (99.3%)	128 (95.5%)	0.002	375 (100%)	186 (95.4%)	<0.001
Yes	3 (0.7%)	6 (4.5%)		0 (0%)	9 (4.6%)	
Pre-pregnancy BMI (kg/m^2)	22.0 ± 3.1	24.0 ± 4.3	<0.001	21.5 ± 2.7	24.4 ± 4.1	<0.001
SBP (mmHg)	117 ± 11	118 ± 15	0.400	116 ± 10	119 ± 14	0.002
DBP (mmHg)	70 ± 9	72 ± 9	0.048	69 ± 8	72 ± 9	0.001
FBG (mmol/L)	4.60 ± 0.33	4.61 ± 0.39	0.754	4.60 ± 0.34	4.60 ± 0.36	0.914
ALT (U/L)	13.0 (10.0–19.0)	13.0 (10.0–21.0)	0.752	13.0 (10.0–19.0)	13.0 (10.0–20.0)	0.485
Cr (mmol/L)	44 ± 8	45 ± 7	0.140	43 ± 8	47 ± 8	<0.001
UA (μmol/L)	212 (189–229)	216 (187–238)	0.021	209 (189–229)	216 (189–241)	0.002
TG (mmol/L)	1.68 (1.29–2.33)	1.90 (1.62–2.62)	<0.001	1.61 (1.24–2.10)	2.16 (1.59–2.87)	<0.001
CH (mmol/L)	4.94 ± 0.99	4.78 ± 0.92	0.144	4.80 ± 0.96	5.07 ± 0.98	0.003
HDL (mmol/L)	1.80 ± 0.37	1.83 ± 0.41	0.137	1.80 ± 0.38	1.82 ± 0.38	0.578
LDL (mmol/L)	2.45 ± 0.77	2.69 ± 0.78	0.002	2.37 ± 0.73	2.77 ± 0.85	<0.001
WBC (×10^9/L)	8.67 ± 2.09	9.10 ± 2.08	0.040	8.50 ± 2.04	9.30 ± 2.09	<0.001
Neutrophils (×10^9/L)	6.04 ± 1.64	6.94 ± 1.95	<0.001	5.84 ± 1.51	7.04 ± 1.92	<0.001
Lymphocyte (×10^9/L)	1.73 ± 0.48	1.66 ± 0.42	0.144	1.71 ± 0.49	1.73 ± 0.43	0.642
Monocyte (×10^9/L)	0.50 ± 0.23	0.47 ± 0.14	0.045	0.51 ± 0.25	0.46 ± 0.13	<0.001
Platelet (×10^9/L)	219 ± 50	232 ± 55	0.010	215 ± 50	236 ± 51	<0.001
Hemoglobin (g/L)	122 ± 11	122 ± 11	0.577	121 ± 11	123 ± 12	0.052

Data are mean ± SD or n (%).

ALT, alanine aminotransferase; BMI, body mass index; CH, cholesterol; Cr, creatinine; SBP, systolic blood pressure; DBP, diastolic blood pressure; FBG, fasting blood glucose; GDM, gestational diabetes mellitus; HDL-C, high-density li*p*oprotein cholesterol; LDL-C, low-density lipoprotein cholesterol; TG, triglycerides; UA, uric acid; DM: diabetes mellitus.

Supplementary Table S3 Characteristics of the External Validation Set.

Variable	Women without GDM	Women with GDM	*P*	Low-risk participants	High-risk participants	*P*
n	1196	409		787	818	
Age (years)	28 ± 4	32 ± 4	<0.001	28 ± 4	30 ± 4	<0.001
Parity (n, %)						
Nulliparous	746 (62.4%)	201 (49.1%)	<0.001	435 (55.3%)	512 (62.6%)	0.003
Parous	450 (37.6%)	208 (50.9%)		352 (44.7%)	306 (37.4%)	
Family history of DM (n, %)						
No	1155 (96.6%)	354 (86.6%)	0.002	779 (99.0%)	730 (89.2%)	<0.001
Yes	41 (3.4%)	55 (13.4%)		8 (1%)	88 (10.8%)	
Pre-pregnancy BMI (kg/m^2)	21.2 ± 3.4	25.5 ± 5.0	<0.001	20.6 ± 3.0	24.0 ± 4.7	<0.001
SBP (mmHg)	112 ± 8	114 ± 13	<0.001	112 ± 8	113 ± 10	0.013
DBP (mmHg)	71 ± 8	72 ± 10	0.060	71 ± 7	72 ± 9	0.034
FBG (mmol/L)	4.40 ± 0.34	4.38 ± 0.39	0.151	4.39 ± 0.35	4.41 ± 0.34	0.314
ALT (U/L)	13.0 (9.6–19.8)	13.5 (10.1–20.6)	0.319	13.0 (9.6–19.3)	13.3 (10.0–20.6)	0.148
Cr (mmol/L)	45 ± 8	46 ± 8	0.006	44 ± 8	46 ± 9	<0.001
UA (μmol/L)	212 (189–238)	278 (232–327)	<0.001	212 (186–240)	233 (201–292)	<0.001
TG (mmol/L)	1.96 (1.38–2.73)	2.40 (1.87–3.04)	<0.001	1.82 (1.28–2.30)	2.40 (1.83–3.35)	<0.001
CH (mmol/L)	5.27 ± 1.22	5.44 ± 1.21	0.013	5.24 ± 1.21	5.38 ± 1.23	0.015
HDL (mmol/L)	1.76 ± 0.43	1.66 ± 0.41	<0.001	1.73 ± 0.43	1.74 ± 0.41	0.488
LDL (mmol/L)	2.62 ± 0.72	3.16 ± 0.91	<0.001	2.43 ± 0.59	3.08 ± 0.85	<0.001
WBC (×10^9/L)	8.39 ± 2.39	9.18 ± 1.98	0.040	8.19 ± 2.28	8.98 ± 2.28	<0.001
Neutrophils (×10^9/L)	5.89 ± 1.94	6.66 ± 1.76	<0.001	5.53 ± 1.75	6.63 ± 1.93	<0.001
Lymphocyte (×10^9/L)	1.82 ± 0.79	1.87 ± 0.60	0.169	1.90 ± 0.83	1.767 ± 0.66	0.001
Monocyte (×10^9/L)	0.55 ± 0.22	0.52 ± 0.18	0.010	0.58 ± 0.24	0.50 ± 0.16	<0.001
Platelet (×10^9/L)	238 ± 65	239 ± 60	0.767	239 ± 65	239 ± 62	0.966
Hemoglobin (g/L)	121 ± 13	122 ± 17	0.129	122 ± 13	121 ± 15	0.051

Data are mean ± SD or n (%).

ALT, alanine aminotransferase; BMI, body mass index; CH, cholesterol; Cr, creatinine; SBP, systolic blood pressure; DBP, diastolic blood pressure; FBG, fasting blood glucose; GDM, gestational diabetes mellitus; HDL-C, high-density li*p*oprotein cholesterol; LDL-C, low-density lipoprotein cholesterol; TG, triglycerides; UA, uric acid; DM: diabetes mellitus.

Supplementary Table S4 Comparison of subjects in the high-risk group and the low-risk group.

Group	Training Set		Internal Validation Set		External Validation Set	
	Low-risk group	High-risk group	Low-risk group	High-risk group	Low-risk group	High-risk group
Women without GDM	721 (89.1%)	225 (50.6%)	335 (89.3%)	101 (51.8%)	731 (92.9%)	465 (56.8%)
Women with GDM	88 (10.9%)	220 (49.4%)	40 (10.7%)	94 (48.2%)	56 (7.1%)	353 (43.2%)

Supplementary Table S5 Major packages of R software used in this study.

Functions	R package
Import and export data	foreign
logistic regression analysis	glm
Plot the receiver operating curve (ROC) and measure the area under the ROC (AUC)	pROC
Plot calibration curves, Brier score and develop nomogram	rms
Decision curve analysis (DCA)	rmda
Plot bar diagrams	ggplot2
Random Forest Algorithm	randomForest and *caret*

Supplementary Table S6 Major results of machine learning (Random Forest Algorithm).

Model Evaluation Metrics	Value
Accuracy	0.814 (0.779, 0.846)
Kappa	0.4141
Mcnemar's Test P-Value	<0.001
Sensitivity	0.9704
Specificity	0.3706
Pos Pred Value	0.8137
Neg Pred Value	0.8154
Prevalence	0.7391
Detection Rate	0.7172
Detection Prevalence	0.8814
Balanced Accuracy	0.6705
Error Rate	19.04%

Supplementary Table S7 Variable importance ranking.

Variable	Mean Gini index
Age (years)	38.67632
Neutrophils ($\times 10^9$/L)	34.69094
Monocyte ($\times 10^9$/L)	26.89689
Hemoglobin (g/L)	28.53494
Platelet ($\times 10^9$/L)	27.05850
ALT (U/L)	28.68929
Cr (mmol/L)	32.81895
UA (mmol/L)	40.38951
TG (mmol/L)	43.68183
HDL (mmol/L)	25.75149
LDL (mmol/L)	36.78405
DBP (mmHg)	25.20617
Family history of DM (n, %)	8.01773
Pre-pregnancy BMI (kg/m^2)	46.23283
Parity (n, %)	16.45382

Mean Gini index: Mean variable importance index over 100 computations.

ALT, alanine aminotransferase; BMI, body mass index; CH, cholesterol; Cr, creatinine; SBP, systolic blood pressure; DBP, diastolic blood pressure; FBG, fasting blood glucose; GDM, gestational diabetes mellitus; HDL-C, high-density lipoprotein cholesterol; LDL-C, low-density lipoprotein cholesterol; TG, triglycerides; UA, uric acid; DM: diabetes mellitus.
